# Difficulties of describing suicide statistics in an international environment: Towards a new European solution?

**DOI:** 10.1192/j.eurpsy.2026.10695

**Published:** 2026-07-31

**Authors:** A. Brezina, J. Lopez-Castromán

**Affiliations:** 1 Independent Researcher, Dillingen, Germany; 2 Department of Psychiatry, University of Santiago de Compostela, A Coruña, Spain

## Abstract

**Introduction:**

In our previous work (European Psychiatry. 2025;68(S1):S1161-S1161), we highlighted challenges in suicide statistics in an international context, using Luxembourg as an example. With a population below 680,000, the country attracts around 200,000 daily cross-border workers. These workers may die by suicide either in Luxembourg or their country of residence, while Luxembourg residents may also die by suicide abroad. Assessing the impact of these demographics is hindered by reporting issues: about 6.7% of deaths of Luxembourgish residents occur abroad and are included in mortality counts, yet their causes are not always reported back. Conversely, around 3.7% of suicides in Luxembourg involve non-residents, but data do not distinguish between commuters and travelers.

This issue extends beyond Luxembourg. Across the EU, an estimated 1.8 million residents work as cross-border commuters, yet most countries lack complete data on the causes of death of their citizens when they die abroad. In a united Europe, this raises a critical question: how does this reality distort suicide statistics? Failure to account for suicide risk in minority or transnational groups limits our prevention strategies.

In 2025, the European Union adopted a regulation establishing the European Health Data Space (EHDS) (Regulation (EU) 2025/327, 2025), which will enable access to patient data across the EU for care and research from 2027 onward.

**Objectives:**

To explore whether the EHDS could streamline cross-border reporting of causes of death for epidemiological and research purposes.

**Methods:**

We analyzed data from the Luxembourg Ministry of Health, the National Statistics Institute, and Regulation n° 2025/327 on the EHDS.

**Results:**

The EHDS will provide research access and explicitly includes death registries among eligible data sources. However, no centralized European database is foreseen. Instead, the regulation focuses on creating interoperable electronic patient files accessible across the EU. It remains unclear whether “cause of death” will be treated as part of medical history or classified within civil registration, which may restrict systematic cross-border data use.

**Conclusions:**

While the EHDS represents a major step toward cross-border medical data exchange, it is not yet designed to ensure accurate classification of transnational deaths, nor to integrate this information automatically. Nonetheless, the regulation allows for the future inclusion of additional categories of patient data. Advocacy is therefore needed to ensure that suicide deaths are counted consistently, regardless of where they occur.

**Disclosure of Interest:**

None Declared

